# Plant Ferritin—A Source of Iron to Prevent Its Deficiency

**DOI:** 10.3390/nu7021184

**Published:** 2015-02-12

**Authors:** Magdalena Zielińska-Dawidziak

**Affiliations:** Department of Food Biochemistry and Analysis, Faculty of Food Science and Nutrition, Poznań University of Life Sciences, 60-623 Poznań, Poland; E-Mail: mzd@up.poznan.pl; Tel./Fax: +48-61-8487352

**Keywords:** ferritin, biofortified plants, bioactive food, iron

## Abstract

Iron deficiency anemia affects a significant part of the human population. Due to the unique properties of plant ferritin, food enrichment with ferritin iron seems to be a promising strategy to prevent this malnutrition problem. This protein captures huge amounts of iron ions inside the apoferritin shell and isolates them from the environment. Thus, this iron form does not induce oxidative change in food and reduces the risk of gastric problems in consumers. Bioavailability of ferritin in human and animal studies is high and the mechanism of absorption via endocytosis has been confirmed in cultured cells. Legume seeds are a traditional source of plant ferritin. However, even if the percentage of ferritin iron in these seeds is high, its concentration is not sufficient for food fortification. Thus, edible plants have been biofortified in iron for many years. Plants overexpressing ferritin may find applications in the development of bioactive food. A crucial achievement would be to develop technologies warranting stability of ferritin in food and the digestive tract.

## 1. Introduction

Iron deficiency (ID) affects a large part of the human population, although adult people in developed countries usually obtain proper amounts of iron in their diet. It is a common malnutrition disorder and causes almost 50% of anemia cases, affecting more than 2 billion people worldwide [1,2]. Even mild and moderate ID, lower than those that cause iron deficiency anemia (IDA), result in impaired human functioning [1]. Because of that, the terms anemia, IDA and ID are used interchangeably relatively often in non-medical literature, even though their meaning is not identical.

The high-risk group of IDA and ID includes infants, young children, teenagers, premenopausal and especially pregnant women [2,3,4,5]. The recommended intake of iron is highly variable and dependent on age, gender and health status of the individual [6]. However, some sociodemographic factors, such as the race/ethnicity, socioeconomic status, religion, dietary habits, *etc.*, also have some significance in the risk of anemia development [7,8].

The ID and IDA have undesired functional consequences, especially in children. They impair the cognitive performance, behavior and physical growth of infants, preschool and school-aged children. Because of the decreased amount of oxygen transported to muscles they change the use of the energy source, which affects the physical capacity and work performance of individuals. Increased morbidity from infections resulted from reduced immunity is observed in the entire human population. Additionally, ID disturbs functioning of the digestive system. During pregnancy the IDA increases perinatal risks both for mothers and their children, as well as leads to increased infant mortality [1].

The biochemical consequences observed in the ID affect the production and metabolism of some neurotransmitters, thyroid hormones, DNA replication and iron-dependent enzymes activity [1].

Due to such a huge impact of ID and IDA on human health and their high incidence, international organizations, led by the World Health Organization (WHO), have developed a number of programs to prevent them.

## 2. Iron in Food—Forms, Bioavailability and Toxicity

Generally, food is a good source of iron and for healthy organisms, without metabolic disorders, an appropriate balance of a diet is not difficult to attain. In developed countries iron deficiency is rarely observed as an isolated disease and is often a consequence of malabsorptive disorders (enteropathies), blood loss [9] and also conscious consumption of a diet with the restricted supply of nutrients (such as improperly planned vegan or slimming diets).

The various forms of iron present in food differ in their bioavailability. The heme iron in the center of hemoglobin and myoglobin molecules has the highest bioavailability and its best sources are meat, fish and seafood. It contributes usually about 10%–15% of daily intake of iron. The absorption of the heme iron reaches 15%–35% and is little dependent on the presence of inhibitors in the consumed food [10,11]. However, even in meat the heme iron is only a part of the total pool of this microelement.

The best sources of non-heme iron are seeds, grains, nuts and the dark green parts of leafy vegetables [11]. Non-heme iron is present in different chemical forms, which significantly affects its absorption, typically reaching a rate of 2%–20% [11]. There are both organic and inorganic compounds. The most common forms of non-heme iron present in food are low molecular weight compounds such as ferric citrate, phosphate, phytate, oxalate and hydroxide, and high molecular weight compounds, *i.e.*, ferritin (protein linking thousands of ferric ions in the mineral core) and lactoferrin in infant formulas (iron transporting proteins). In plant food fortification and supplementation iron salts are also usually used, such as ferrous sulfate, ferrous gluconate, ferric chloride and ferric EDTA, and less commonly iron carbonyl, amino acid chelate and iron-dextran (ferric iron linked by oxygen atoms with a polysaccharide) [12,13,14].

There is a powerful regulation mechanism of iron absorption in the human body. It is the consequence of a lack of a physiologic mechanism for iron excretion. The absorption of iron strongly depends on the organism iron status. Iron uptake usually increases 10-fold in the case of its deficiency and this rule applies both to heme and non-heme iron. Moreover, the absorption of non-heme iron is influenced both by its inhibitors (phytate, polyphenols, calcium, milk and egg proteins, albumins) [10] and enhancers, such as ascorbic acid and muscle tissue proteins [13].

The major transport of heme iron takes place in duodenal enterocytes and hepatocytes. Intestinal uptake occurs via a saturable, carrier-mediated process, through the HCP1 transporter (heme carrier protein 1, also known as the proton-coupled folate transporter (PCFT)) [15]. Recently, two other heme transporters were identified in mammalian cells: FLVCR (feline leukemia virus subgroup C receptor) [16] and BCRP (breast cancer resistance protein) [17]. They are efflux protein; however, their role in intestinal iron uptake has not been clarified [16,17].

It has been assumed for many years that the uptake of non-heme iron through enterocytes depends only on the divalent metal transporter 1 (*DMT1*) [13,18]. However, it is not a universal receptor for non-heme iron ions. Now it has been found that soybean ferritin is absorbed via mu2 (AP2)-dependent (assembly protein 2 complex subunit mu) endocytosis [19] and lactoferrin with LfR (lactoferrin receptor, expressed with high efficiency in infants) [20].

Occasionally, in spite of the existing regulatory mechanisms, iron overload states may occur. The metabolic disorder associated with an excess of iron in the body is known as haemochromatosis. This genetic disorder affects about 0.25%–0.5% of the Caucasoid population [21]. However, some deaths have also been reported as a result of overdose of preparations containing high iron levels (36–443 mg iron/kg body weight) [22]. Toxicity of iron is a consequence of its role in the formation of hydroxyl radicals in the Fenton reaction. Thus, both U.S. Food and Drug Administration and the European Commission imposed regulations concerning iron content and labeling of its supplements and supplemented food [23,24]. Maximum recommended intake levels have also been established for this microelement. The amount of iron consumed with food or supplements should not exceed 40 mg per day for children up to 13 years of age and 45 mg for the rest of the population. These rules of course apply to healthy people, with average physical activity levels [6].

## 3. Food Enrichment with Iron

Due to the prevalence of iron deficiency many programs have been initiated, such as food supplementation, fortification or modification of food processing. One of the strategies for iron deficiency replenishment is the use of dietary supplements. They are multivitamin and multimineral preparations or iron-only supplements and contain mainly ferrous and ferric iron salts [25]. Other forms include heme iron polypeptides, iron chelated with amino acids and complexed with polysaccharides [22].

However, the WHO [26] recommends food fortification as a way to increase iron intake. Many food products are fortified with this microelement in different doses, even up to 100% of the estimated average requirement. Ferrous sulfate, ferrous fumarate, ferric pyrophosphate and electrolytic iron powder are admissible in food fortification.

Cereal products are commonly fortified with iron and it is estimated that as much as 25% of total daily intake in Sweden and the United States come from fortified food [27]. However, many of the products are enriched with not recommended low-cost elemental iron powders, with very low bioavailability, even down to 15% of the native food iron [10,27]. Many food products are also sensitive to color or flavor changes and oxidative damage of some nutrients [28].

It is well documented that iron supplementation of the human diet, especially with its higher doses, brings a health risk, causing some gastric problems such as gastric upset, abdominal pain, vomiting, faintness, constipation and nausea [9,10,25]. Iron introduced to the diet may also lead to oxidative damage of some cellular components, when antioxidant compounds are not delivered [29].

Thus, alternatives to food fortification are sought after. It is considered that the nutritional value of plant origin foods increased through biofortification will provide a more efficient and sustainable solution to the problem of iron deficiency [30].

The main strategy of plant iron biofortification is an enrichment of legumes and cereals with ferritin iron. Ferritin concentration in edible parts of plants varies greatly. It was suggested that its concentration in beans or seeds is about 50–70 mg/kg, which corresponds to the iron content of 10 mg/kg [31]. This means that intake of 100 g fresh, raw bean/seeds would provide only 12.5% of recommended daily allowance for non-vegetarian adult men or 6.66% for non-vegetarian women aged between 19–50 years. This concentration is too small to guarantee a significant dietary iron supply, thus, it is necessary to find a method to increase the ferritin iron content and use this vegetable protein with unique properties to control iron deficiency anemia.

## 4. Specific Features of Ferritin

Ferritin is a protein occurring in almost all living organisms (except yeasts) [32] in a very conservative spherical structure. In higher plants and animals even the primary sequence is conserved. The molecule is the protein apoferritin coat, often called shell, filled with an iron core. The coat is usually formed from 24 structurally equivalent polypeptide subunits with molecular weight of about ~20 kDa in mammals [33] and ~25–28 kDa in plants [34]. Mini-ferritin, composed of 12 subunits, is present in bacteria [13].

Each of these polypeptide subunits forms four long helixes, combined in pairs. They create a hollow protein shell with the outside diameter of 12–13 nm and inside diameter of 7–8 nm. The protein has an octahedral symmetry [35]. Inside the polypeptide chain a conservative ferroxidase center is present and its activity determines the ability of ferritin to oxidize iron ions from Fe(II) to less toxic Fe(III). Iron as a ferric oxyhydroxide is bound in the combination with varying amounts of phosphates. The content of phosphates is higher for plant than for animal ferritin [30]. Moreover, heme-containing ferritin is synthesized by bacteria [36].

Prokaryotes synthesize three types of ferritin: the dodecameric miniferritin (Dps), filled with ~500 atoms of Fe(III he bacterioferritins (BFR) accumulating ~1800 Fe(III) ions or 12 heme molecules, and the bacterial ferritins (FTN), similar to the H-subunit of human ferritin, which capture up to 2500 Fe(III) atoms [33].

In vertebrates ferritin is composed of subunits of two different types: heavy (H) with the molecular weight ~21 kDa, and light (L) about ~19.5 kDa. The ferroxidase center, responsible for iron binding and oxidation of the ferrous iron, is a part of the H-subunit. The L-subunits contain nucleation sites important during the slower iron oxidation and mineralization [35]. It accommodates up to 4500 iron ions.

Plant ferritin—phytoferritin—is similar to the animal variant both in its function and structure. It is composed of H subunits, which share 40% of amino acids sequence identity with the animal H equivalent [37]. Simultaneously, the functions of the plant H subunits are similar both to the animal H-subunit (because they contain the ferroxidase center) and to the L-subunit (due to the amino acid residues responsible for iron nucleation) [35,38].

To date, almost all known, naturally occurring seed phytoferritins consist of two H subunits, with molecular weights of 26.5 (H1 subunit) and 28 kDa (H2 one). Their sequence identity is high (e.g., 82% for soybean ferritin) and initially it was considered that the H1 subunit is generated during degradation of the H2 subunit, induced by hydroxyl radicals [39,40]. Currently it has been proven that they are encoded by distinct genes [37,41]. The ratio of these two subunits depends on the phytoferritin source [42] and influences the protein stability [43].

The plant protein subunits are synthesized in a precursor 32 kDa form, which consists of the fragment responsible for subcellular targeting—transit peptide (TP), and the other—extension peptide (EP)—involved in protein stability control during iron exchange [44,45,46,47]. When the plant ferritin enters the plastid, the TP is cleaved from the *N*-terminus of the plant ferritin sequence [48]. The mature form of plant ferritin retains in its structure 24 EP fragments composed of 30 amino acids residues, which stabilize the oligomeric conformation of ferritin [44,45,46,47].

The role of ferritin in cells is to provide iron concentration at the level necessary to their proper functioning (10^−3^–10^−5^ M). This unique molecule has the ability to accumulate iron up to the 10^−2^ M, while the solubility of Fe(III) under physiological conditions of pH, temperature and the presence of air is only 10^−18^ M [34,48]. Usually the iron content in the molecule is below 3000 atoms [49], even if a higher accumulation may occur (up to 4500 atoms). Such a high concentration of iron ions in ferritin is possible as a result of the high density of the iron core, 2.5 times higher than that of the polypeptide shell. The volume of the core is only one quarter of the whole molecule. Then, the molecular weight of the protein, which for apoferritin is ~480 kDa, is almost doubled when it is filled with iron [49].

Most of the cellular iron is stored in ferritin, which is both the acceptor and donor of iron for metabolic processes. This protein also regulates the cellular concentration of transition metals [50], metal ions apart from iron in the mineral core (including beryllium, aluminum, zinc, cadmium and lead). This confirms its function in cell detoxification [51,52,53,54,55,56], limiting the formation of reactive oxygen species, and mitigating their effect on cellular structures and macromolecules (lipids, proteins, DNA) [57]. Thus, by capturing toxic metal ions, ferritin is simultaneously involved in the antioxidant system of defense [58].

Moreover, bacterial miniferritin—*Dps* ferritin—protects DNA against nucleases and free radicals binding that molecule. In a consequence of DNA binding, Dps ferritin inhibits the level of gene expression. This form of ferritin is induced not only in response to H_2_O_2_ formation, but also during starvation (e.g., carbon starvation) [13,33,36]. Nuclear ferritin present in animal cells plays not only a key role in protecting DNA against oxidative damage. It also regulates iron accessibility to the component of the nucleus; moreover, it is a transcriptional factor [59,60].

Phytoferritin also plays an important role in the defense against pathogen attacks. Overexpression of ferritin as a result of biotic stress induces complexing of iron circulating in host organism—iron becomes inaccessible to pathogens’ life processes [33,57,61].

While the animal ferritin is present in the cytoplasm, phytoferritin is unevenly distributed in plant organelles, primarily in the plastids—non-green plastids in leaves, or amyloplasts in roots and seeds. In response to stress it appears in chloroplasts. Legumes also possess the ability to synthesize ferritin in root nodules [62,63,64]. Phytoferritin has never been found in the cytoplasm, but it may exist in mitochondria [65]. Its synthesis is regulated both on the transcription and translation levels, in response to the iron presence or stress conditions, especially oxidative stress [33]. It is considered that root iron absorption systems as well as the regulation of ferritin expression serve to maintain correct iron concentration at the cellular and the whole plant level [66]. Iron is transported to leaves, where it is complexed with ferritin. Ferritin becomes the iron source used for the synthesis of Fe-containing protein necessary for photosynthesis, during the development of the photosynthetic organs after the process of germination [39].

Among vegetative organs of plants, leaves accumulate most of iron, with ferritin synthesis in leaves increasing during their development. Roots, even if they uptake iron from the soil, accumulate less iron than leaves; however, the symbiotic root structures specific for legume plants, *i.e.*, nodules, are rich in ferritin, especially in the early stage of their development. Significant amounts of ferritin are accumulated in seeds, as a consequence of its remobilization from vegetative organs: even 20%–30% from leaves [67,68] and 40%–60% from nodules, as it was observed for soybean [69]. The presence of ferritin as a main source of iron in the grain and seeds proves that it is a long-term iron storage protein. Due to this reutilization of nodule iron, legume seeds (soybean, chick peas, lentils, lupine) are especially rich in ferritin, in contrast to cereal grain, such as wheat or rice [69].

Because of the efficient ferritin expression in legume seeds, a new role and function of this plant protein has been increasingly more often emphasized nowadays, *i.e.*, its potential use in food fortification.

## 5. Nutritional Value of Plant Ferritin

Experiments on ferritin bioavailability have been conducted on a large scale and they are focused especially on legume proteins. In the beginning the results were unsatisfactory, but they were regarded as flawed ones [70], especially because of the problem with soybean ferritin iron labeling. Subsequent research suggested high bioavailability of soy ferritin, comparable to the bioavailability of FeSO_4_, both during *in vitro* experiments [19,71], in trials conducted on rats [72,73] and in clinical studies [12,74,75,76].

Stability of ferritin is still hotly disputed. Many *in vitro* studies confirmed the instability of ferritin during digestion. It appears to be inconsistent with those experiments conducted *in vivo* in humans, confirming the good bioavailability, stability and a different mechanism of ferritin iron absorption than that for Fe(II) ions [12,73,74,75,76,77].

The ferritin iron was released from the ferritin protein shell of pea seeds after cooking and during digestion in the artificial stomach [78]. Another *in vitro* study suggested also that the ferritin iron after digestion is absorbed as non-heme iron, while ascorbic and phytic acids influence this process [79]. However, these results totally disagree with most of the clinical studies. Theil *et al.* (2012) [77] presented competition studies between FeSO_4_ and the ferritin iron. Even a nine-fold excess of FeSO_4_ in the diet did not influence absorption of the ferritin iron by humans (4.5 mg of FeSO_4_ and 0.5 mg of labeled ferritin iron ^59^Fe)—the ferritin iron bioavailability from a such composed iron source was the same as it was from the diet containing only the ferritin iron. This suggests that these two types of iron do not compete for the same receptor (DMT1) and are absorbed through different absorption mechanisms.

The mechanism of ferritin uptake was examined in Caco-2 cells, which have been established as a good model of experiments on nutrients absorption in the small intestine. It was proven that soybean ferritin is absorbed through endocytosis dependent on assembly peptide 2 (AP2) [19]. The effect of iron absorption inhibitors was also examined in that model. The results are consistent with those obtained *in vivo*. Calcium and phytic acid had no effect on the absorption of iron from ferritin, while tannic acid even increased it [71].

The still observed discrepancy between *in vitro* and *in vivo* results may have some evident reasons, the most important of which is that *in vitro* experiments are carried out on isolated and purified ferritin, because of low ferritin concentrations in plants [78,79]. Thus, the influence of the food matrix on ferritin stability is not taken into consideration. Moreover, *in vitro* digestion relatively often is more efficient than *in vivo* digestion*.* Finally, the instability of ferritin under strongly acidic conditions and re-shaping the structure at higher pH values were observed [43]. Refolding of ferritin at higher pH was even used to encapsulate unstable nutrients [80]. Thus, the process of ferritin refolding with the iron core could be possible also in the small intestine.

The thesis that ferritin at least partially survives digestion *in vivo* was supported by the results of experiments on encapsulated ferritin absorption in human organisms. Ferritin was encapsulated both in polymers resistant to acidic pH of the stomach (gelatin coated with Eudragit), and in polymer non-resistant to digestion (gelatin). No differences in the absorption of these two forms of the ferritin iron were observed, so protection against the acidic environment of the stomach was unnecessary [77].

Thus, summing up, we may expect the following mechanisms of ferritin digestibility and absorption in the human digestive tract. The first—the most optimistic option—implies that ferritin survives digestion and is fully absorbed *via* endocytosis. However, if the ferritin protein shell is opened during digestion (through proteolysis or denaturation), absorption of iron from the core may take place in two different ways: the iron mineral insoluble core may be entirely absorbed, or iron from the mineral core could be released and absorbed as the ferrous iron by divalent metal transporter 1 (DMT1) [81]. Finally, ferritin may be denatured in the stomach and release the iron and then it may refold in the small intestine, capturing the iron core again, as it was observed during the encapsulating of carotene [80]. A combination of these mechanisms may be expected as well. However, it should be remembered that ferritin stability may be greatly influenced by food matrix digestibility. Isolated ferritin, deprived of the protective action of other food ingredients, may be more susceptible to degradation.

## 6. Iron Biofortification of Crop Plants

A purposeful increase of microelement or vitamin contents through breeding or biotechnology is defined as plant biofortification. Iron is an essential element for plant development and mechanisms of its absorption and accumulation are exploited for biofortification processes. Three different methods may be applied to achieve this: (1) creating and searching for some new cultivars through conventional breeding and selection techniques; (2) creating plants with desired properties by genetic engineering; or (3) simple iron enrichment of plants by increasing the supply of this micronutrient in the soil or culture medium.

Many studies are focused on the creation of transgenic plants, rich in iron. Their generation is based on different strategies. The first used strategy leads to increased contents of molecules storing and/or complexing iron through the expression of the soybean ferritin gene [82,83,84,85], human lactoferrin gene [86], genes responsible for phytosiderophore biosynthesis [87,88,89], or the soybean leghemoglobin gene [90].

The second strategy is based on the expression of different micronutrient transporters. The first group of these transporters enhances the uptake of iron from the soil **(**e.g., OsIRT1, OsNRAMP1, OsNRAMP, PEZ 1 and PEZ 2). Unfortunately, these transporters are not selective to iron only, so even if a higher concentration of iron in plants was obtained, the concentration of toxic cadmium also increased significantly [91,92]. Some success was achieved in manipulating the expression of the membrane transporter responsible for the direction of iron to seeds (Fe(II)-NA transporter) [93] or knock-down expression of the Fe vacuolar transporter [94].

Another strategy relies on the reduction of contents of iron absorption inhibitors, *i.e.*, phytic acids [95,96], or on increasing activity of enzymes, which degrade them, such as phytase [97,98]. Some studies have also been conducted on biofortification of food with iron uptake enhancers, such as insertion genes of nicotianamine synthase [99,100,101,102] or metallothionein-like proteins [32,98,103].

The latter of the most important transgenic approaches leads to the overexpression of the Fe homeostasis-related transcription factor [104,105].

The presented strategies may of course be used in different combinations to enhance the success of biofortification, *i.e.*, efficiency of iron content increase in plants. Usually modification of ferritin expression is combined with the expression of other genes [106,107,108].

Huge progress in the overexpression of ferritin in rice has been observed since the time when the first attempts were made on rice biofortification with iron [82,83]. Usually soybean ferritin genes under the control of endosperm-specific promoters were used, and typically the iron concentration increased two to four times at a single introduction of ferritin gene [82,84,98,109]. A combination of different genetic modification approaches yielded better results, e.g., the introduction of the ferritin gene under the control of endosperm-specific promoters, the nicotianamine synthase gene and the gene of Fe(II)-nicotianamine transporter OsYSL2 (under the control of an endosperm-specific promoter and the sucrose transporter promoter) increased iron concentration in polished rice seeds six-fold [110].

Important problems in these experiments were related to the accumulation of ferritin in the husk, leading to significant amounts of iron being lost in polished seeds. Moreover, the overexpression of ferritin is not synonymous with a high increase in iron content. This problem was observed in a new transgenic basmati rice line, which overexpressed rice ferritin. Even if ferritin content was 7.8-fold higher, the grains accumulated only 1.4-fold more Fe when compared to wild type rice [84]. Very efficient “hyperexpression” of ferritin was obtained in a rice line created with the soybean ferritin gene under the control of two strong promoters, OsGlb1 and OsGluB1. However, even if ferritin concentration increased 13 times, iron content was only ~30% bigger [85].

Obviously not only rice has been subjected to genetic manipulation. Some experiments were conducted on other edible plants, such as maize [51,111,112,113], wheat [112,113] and lettuce [114]. The same problems have appeared, such as a problem with ferritin transport to grains or a disproportion between the increase in the iron contents and the level of ferritin overexpression.

Commercialization of crops biofortified in micronutrients meets different limitations, at first a lack of acceptance by farmers (iron enriched plants may be degenerated, discolored), and by consumers (because of the price and concerns over genetically modified food). Thus, the methods based on selecting varieties richest in iron (beans, lentils) [115,116], using fertilizers or native gene overexpression, continue to be very popular and attractive.

The use of specially composed fertilizers seemed to be an apparently easy method for plant biofortification. However, the correlation between iron concentration in the soil and in cereal grains or legume seeds is far from linear. Moreover, significant changes in the appearance of plants were observed. They became discolored and dwarfed, while plant yields strongly decreased and edible plant parts were unattractive to the consumer; moreover, the method was not sufficiently effective [117,118].

Thus, a concept for the use of legume sprouts growing under special conditions was proposed. Due to the efficient uptake of ferrous iron by plant roots during sprout cultivation in some FeSO_4_ solutions overexpression of ferritin was observed. The accumulation of iron in these sprouted seeds was high, proportional to the concentration of Fe(II) ions in the culture medium. Even if produced sprouts were thickened, shortened and discolored, ferritin may be effectively isolated from them or the sprouts may be dried, milled and introduced into food in that form. This overexpression was observed both in different legumes and in wheat. The concentration of ferritin iron in soybean sprouts increased almost 70-fold, and more than 18 times in wheat, which accounted respectively more than 60% and 50% of total iron content [119,120]. This accumulation may be even higher if you take into account differences in the tolerance to the presence of iron in the culture medium or soil by varied genera and varieties of legume plants [121].

Due to the nutritional value of ferritin, its increased content in edible plants seems to be a very attractive method to prevent iron deficiency. It may be a cheaper and more effective approach than food supplementation and fortification. Because of the insufficient native ferritin iron concentration even in legume plants [31], the success of its use in the development of food products for special nutritional purposes is dependent on the success in its overexpression.

## 7. Ferritin as a Promising Bioactive Food Ingredient

The unique properties of ferritin presented above and prospects for its effective overexpression may find practical applications in the development of bioactive food, *i.e.*, food designed to modulate some metabolic processes. In this case food could be dedicated to people suffering from iron deficiency anemia.

At first the edible plants rich in ferritin iron may be used solely as a source of iron, regardless of the form in which it is transported to the place of absorption (*i.e.*, to the small intestine).

However, other opportunities will appear if undamaged ferritin may be transferred to the intestine. Endocytosis of ferritin through enterocytes may be a tremendous chance for individuals, whose requirement for iron uptake is increased (e.g., because of blood loss). This mechanism is also promising in the treatment of inflammatory bowel diseases [77,81,122].

What is more, ferritin iron is safer because of its limited contact with the environment. The iron enclosed in the protein shell would not come into contact with food ingredients and/or with the cells of the digestive tract. The food should be protected against oxidative changes or alteration of sensory properties. Moreover, oxidative damages of the intestine cells should also be restricted. The slow iron release from ferritin protects the cells against oxidative damage compared to other iron supplements [13,123].

The development of bioactive food with an increased ferritin iron content encounters other difficulties. Ferritin iron concentration in food of plant origin depends on the botanical genus of the used seeds or grains, but it is also a consequence of crop growing conditions (especially iron concentration in the soil) and food processing. Because ferritin is a protein, food should be processed under conditions that do not cause thermal denaturation. Thus, temperature or time of exposure to high temperatures should be limited. It was shown that ferritin survives some thermal processing, even if the applied temperatures exceed these considered as degrading the protein, *i.e.*, over 80 °C [75,124]. Some non-thermal processes, such as dehulling or protein coagulation, also remove the ferritin iron, so their application should be limited. The pH stability of ferritin, even if relatively high, should also be kept in mind, even if a limited number of traditionally fortified food products have such extreme pH to cause the ferritin denaturation.

Finally, the problem with the analysis of ferritin iron concentration in the designed food needs to be stressed here. Even if ferritin content is high, it does not mean that the ferritin iron survives food processing. Ferritin may be determined with various techniques, mainly immunochemical [13], but iron speciation is necessary. It was repeatedly proven that the contents of ferritin did not reflect the contents of ferritin iron. A lack of a method, which would make it possible to determine the ferritin-iron content quickly and easily may strongly limit progress in the application of phytoferritin in food fortification, since the efficiency of binding iron in the desired form may not be well calculated. Iron saturation of the protein shell varies between phenotypes and genotypes [23,125], while factors that affect the iron density are still unknown [126]. Methods facilitating differentiation of possible iron forms are needed in order to select promising plant varieties and breeding strategies [126].

Hoppler *et al.* (2009 and 2014) [127,128] proposed the method of iron speciation in plant samples. Isotope dilution mass spectrometry (IDMS) of samples spiked with [^57^Fe]-labeled bean ferritin and thermal ionization mass spectrometry, enabled ferritin iron quantification. Another method worth considering is that based on the colorimetric analysis of different iron forms extracted from food samples (organic and inorganic complexes and the ionic form of iron) [129]. However, for some applications, e.g., technological process control in food industry, the first method may be too expensive and, what is more important, time-consuming. For another, the precision of the second method may be not enough. Some new, rapid, cheap and precise analytical techniques should be introduced into the analysis of food enriched with the ferritin iron.

## 8. Conclusions

Due to the presented above, exceptionally promising properties of ferritin and results of *in vivo* experiments on ferritin iron bioavailability, commercial production of nutraceuticals and bioactive food containing this protein may be expected soon. However, more studies are required to comprehend ferritin stability in the human digestive tract and during food processing as well as more *in vivo* studies to understand the mechanism of the protein absorption.
